# The Hospital World

**Published:** 1910-07-02

**Authors:** 


### The Hospital World.

### Elections and Appointments.

Mr. Tom B. Harrison, late assistant to the Secretary Superintendent at Addenbrooke's Hospital, Cambridge, has been appointed to the Secretaryship of the Chichester General Infirmary, at the special meeting of the Board held at Chichester on June 28 last. Mr. Harrison came to Addenbrooke's about five years ago, shortly after Mr. R. J. Coles, the present Secretary Superintendent, took up his appointment, and soon proved himself a highly capable and thoroughly efficient assistant. Before coming to the Cambridge institution he had nine years practical experience in hospital work at the North Staffordshire Infirmary and Eye Hospital, Hartshill. During his term as assistant secretary at Addenbrooke's he has been actively associated with the recent improvements and the various new systems which have been introduced in that up-to-date institution, and has won golden opinions from those who have been in a position to judge of his capabilities. We understand that he was finally chosen from three candidates selected out of a very large number who applied for the vacant post at the Chichester General Infirmary. His wide and varied experience, and the fact that he has taken special interest in infirmary work, will make the new secretary a valuable acquisition to the Chichester institution, and we congratulate both him and the Infirmary upon the choice which the Governors have made.

### Personalia.

Mr. George Bisset and Mr. John Clark have been enrolled as managers for life of the Morningfield Hospital, Aberdeen, in respect of donations by the Northern Co-operative Company, Limited.

The following gentlemen were appointed as House Committee for the ensuing month at the Worcester Infirmary: The Rev. E. J. Boon, the Rev. Father Kenny, Dr. Pollard, Mr. W. Park, and Mr. P. S. Williams; and Father Kenny and Mr. Williams were elected as Visitors.

An appreciation of Mr. Cosmo Bonsor, the well-known treasurer of Guy's Hospital, appears in the current issue of the *City of London Illustrated*. It is illustrated with an excellent photograph of the full-length portrait of Mr. Bonsor, painted by Mr. Herbert Draper for the Governors and staff of Guy's Hospital. This portrait, which is one of the best shown in this year's Academy exhibition, shows the genial treasurer standing in a characteristic attitude, and will be hung in the Court Room at Guy's. Mr. Bonsor, who is a son of the late Mr. Joseph Bonsor, of Polesden, Surrey, was born on September 2, 1848, and is an old Etonian. From the appreciation, to which is added the article on "Guy's Hospital and its Treasurer" that appeared in The Hospital of November 16, 1907, we learn that he has recently become an enthusiastic motorist.

